# HAS YOUR SMILE BEEN CHECKED?: CHARACTERISTICS OF DENTAL CARE UTILIZATION OF MEDICARE BENEFICIARIES

**DOI:** 10.1093/geroni/igad104.2338

**Published:** 2023-12-21

**Authors:** Lillian Agyemang, Louanne Bakk

**Affiliations:** University at Buffalo School of Social Work, Buffalo, New York, United States; University at Buffalo, Buffalo, New York, United States

## Abstract

**Background:**

An area under researched is oral health disparities in the population of older adults residing in the United States. The current dental benefits offered through health insurance plans that older adults are likely to utilize, particularly Medicare and Medicaid, are inadequate. The deficiency harms this population’s physical and mental health and well-being. Additionally, insufficient oral health care has adverse outcomes, including tooth loss, poor self-esteem, malnutrition, and cardiovascular complications. While evidence suggests race and ethnicity can be predisposing factors impacting access to dental care, it is unclear the extent to which disparities in utilization exist.

**Methods:**

This exploratory, cross-sectional study examined the relationship between dental care utilization and Medicare beneficiary characteristics. Data from the 2018 wave of the Health and Retirement Study were used. The sample consisted of 8,357 respondents aged 65 and older enrolled in Medicare.

**Results:**

There was an association between dental care utilization and race, ethnicity, health status, education, and age. A much lower proportion of Black and Hispanic older adults reported seeing a dentist in two years than non-Hispanic White beneficiaries (p<.001). Dental care utilization was also less among respondents who were older (p<.01), in poorer health (p<.001), and had less education (p<.001).

**Conclusions and Implications:**

Findings suggest disparities in dental care utilization exist and disproportionally impact vulnerable Medicare beneficiaries. This is concerning, particularly given racial and ethnic differences in health. The conclusions accentuate areas such as access to dental care services, coverage, and policy implementation concerning and impacting older adults.

